# A Microwave Platform for Reliable and Instant Interconnecting Combined with Microwave-Microfluidic Interdigital Capacitor Chips for Sensing Applications †

**DOI:** 10.3390/s20061687

**Published:** 2020-03-18

**Authors:** Juncheng Bao, Gertjan Maenhout, Tomislav Markovic, Ilja Ocket, Bart Nauwelaers

**Affiliations:** 1 Div. Telemic, Department of Electrical Engineering (ESAT), KU Leuven, Kasteelpark Arenberg 10, Box 2444, 3001 Leuven, Belgium; gertjan.maenhout@kuleuven.be (G.M.); tomislav.markovic@esat.kuleuven.be (T.M.); ilja.ocket@imec.be (I.O.); bart.nauwelaers@esat.kuleuven.be (B.N.); 2 IMEC, Kapeldreef 75, 3001 Heverlee, Belgium

**Keywords:** integrated circuit interconnections, microwave dielectric sensors, biosensors, microfluidics, spectroscopy

## Abstract

This work presents a novel platform conceived as an interconnect box (ICB) that brings high-frequency signals from microwave instruments to consumable lab-on-a-chip devices. The ICB can be connected to instruments with a standard coaxial connector and to consumable chips by introducing a spring-levered interface with elastomer conductive pins. With the spring-system, microwave-microfluidic chips can be mounted reliably on the setup in a couple of seconds. The high-frequency interface within the ICB is protected from the environment by an enclosure having a single slit for mounting the chip. The stability and repeatability of the contact between the ICB and inserted consumable chips are investigated to prove the reliability of the proposed ICB. Given the rapid interconnecting of chips using the proposed ICB, five different interdigital capacitor (IDC) designs having the same sensing area were investigated for dielectric permittivity extraction of liquids. The designed IDCs, embedded in a polydimethylsiloxane (PDMS) channel, were fabricated with a lift-off gold patterning technology on a quartz substrate. Water–Isopropanol (IPA) mixtures with different volume fractions were flushed through the channel over IDCs and sensed based on the measured reflection coefficients. Dielectric permittivity was extracted using permittivity extraction techniques, and fitted permittivity data shows good agreement with literature from 100 to 25 GHz.

## 1. Introduction

In the past two decades, microfluidic technology [1] has provided significant influence on different scientific domains. Parallelization of micro reactors contributed to increase the throughput of various chemical and biological reactions with reduced sample consumption [2,3]. Furthermore, bringing microwave signals to lab-on-a-chip platforms has created a new subdomain called microwave-microfluidics, which has drawn scientific attention in recent years [4]. A broadband spectroscopy technique based on on-wafer coplanar waveguide (CPW) topology has been developed and applied to characterize nL volume samples of deionized (DI) water [5,6,7], human umbilical vein endothelial cells (HUVECs) [8], and a single biological cell [9]. Planar [10,11] and substrate-integrated waveguide (SIW) cavity resonators [12] were developed to sense mixtures of DI water and isopropanol (IPA) with different volume fractions. Moreover, microwave heaters in microfluidics enabled localized, selective, and efficient heating of droplets in continuous and digital microfluidics platforms [13,14,15]. Aside from devices operating in the microwave frequency range, numerous devices operating in the infrared and optical frequency range were developed for microfluidic applications as well. A planar-chalcogenide-waveguide-integrated microfluidic sensor was fabricated [16] to extend electromagnetic microfluidic sensing spectrum to infrared range. In addition, silicon light emitters [17] were integrated with microfluidic channels for various biosensing applications such as rapid fluorescent bioanalysis and environment monitoring [18]. Finally, an optical sensor in phosphorescent iridium (III) complexes was built to monitor oxygen levels in living cells [19].

All these applications were enabled by novel manufacturing technology, which has brought together planar microfluidic devices, planar microwave devices, and standard electrical signal interfacing. This interfacing of high-frequency signals to the lab-on-chip systems is usually done in two ways. One of them is based on probes [5,7] while the other is based on connectors [12,15]. The probe-based approach can provide precise and highly repeatable contacting between the probes and microwave circuits on a chip with miniaturized contacting pads. This makes the calibration of the system accurate, which helps to characterize the dielectric permittivity of unknown liquids in previously mentioned spectroscopy applications. However, the probe-based connection approach has its own drawbacks. First of all, a bulky and expensive probe station is always necessary. This automatically limits the mobility of the experimental setup. Furthermore, special training is mandatory for using the probe-based connecting system and poor operation can easily destroy the probes, which brings extra cost. All these factors limit the broadcasting of the microwave-microfluidic applications to nonmicrowave specialists, such as biologists and chemists. For connector-based connection approach, a connector is mounted on a microwave-microfluidic chip to provide a stable connection between the microwave source and the microwave-microfluidic chip [15]. However, if the chip has to be disposed, the cost of the connector and the effort to mount the connector stacks up to the total cost dramatically. Besides, the connectors need to be carefully torqued each time the microwave-microfluidic chip needs to be changed, which often generates significant efforts once the experiment requires frequent changes of chips.

All above drawbacks are preventing the broadcasting of microwave-microfluidic technology to nonmicrowave researchers or even for commercialization. This encourages us to search for a novel interface between the expensive lab equipment and the relatively cheaper or even consumable microwave-microfluidic chips. In other words, to make widely usable microwave-microfluidic technology, a novel interface is needed. This interface should be cheap, easy to manufacture, and should offer high mobility. Furthermore, it should provide instant and stable contact between instruments and microwave-microfluidic chips.

In this paper, a novel idea of interconnecting microwave-microfluidic chips and microwave instruments is proposed. Firstly, the novel interface is described and validated with microwave measurement. Then, the stability and repeatability of the contacting between the interface is investigated. Finally, given the possibility to rapidly interconnect microwave-microfluidic devices using the novel interface, five different interdigital capacitor (IDC) sensor designs having the same sensing area were investigated for dielectric permittivity extraction. The designed IDCs, embedded in a microfluidic channel built in a polydimethylsiloxane (PDMS) layer, were fabricated with a lift-off gold patterning technology on a quartz substrate. Broadband dielectric permittivity extraction shows a huge potential for the proposed interface to make microwave-microfluidic measurements much more accessible to a broader scientific community. 

## 2. Interconnect Box

### 2.1. Manufacturing

**Figure 1:**
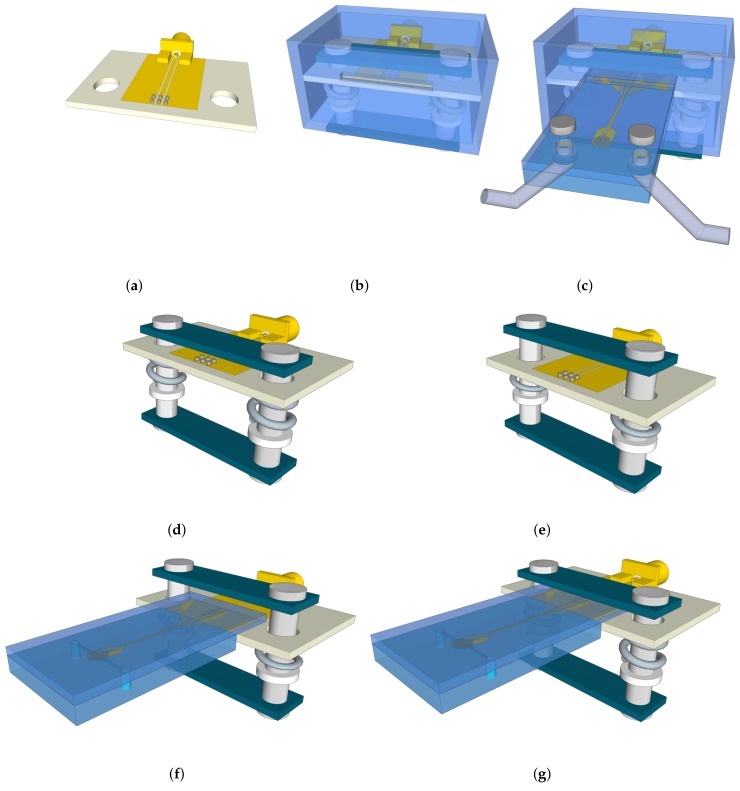
(**a**) A printed circuit board (PCB) with an subminiature type A (SMA) connector and Invisipins. (**b**) A polymethyl methacrylate (PMMA) enclosure with the PCB. (**c**) An interconnect box (ICB) loaded with a consumable microwave-microfluidic chip. (**d**) The initial position for the loading of the chip. (**e**) Compression of springs prior to the loading of the chip. (**f**) Loading of the chip and positioning over Invisipins. (**g**) Releasing of the spring-levered handle and finalizing the loading of the chip.

The interconnect box (ICB) is presented in Figure 1a–c and consists of a polymethyl methacrylate (PMMA) enclosure (Figure 1b) housing a complete electric–mechanic system. This ICB together with a consumable microwave-microfluidic chip results in a novel sensing platform, which is compatible with optical systems (Figure 1c).

The electric part of the system consists of a microwave transmission line (Figure 1a) inside of the PMMA enclosure. The transmission line is manufactured in a CPW topology on a Rogers printed circuit board (PCB), having the right dimensions to achieve a characteristic impedance of 50 Ω. Namely, dimensions of the outer ground electrodes are 5 mm while the central electrode is 0.75 mm wide, altogether spaced for 0.15 mm. As depicted in Figure 1a, a subminiature type A (SMA) connector is located on one side of the CPW transmission line, which is used for interconnecting the PCB with laboratory instruments such as vector network analyzer (VNA), interconnecting cables, power amplifiers, and so on. On the other side of the PCB, Invisipins (RD Interconnect Solutions 5502-14-003) are soldered onto the signal and ground electrodes to conduct high-frequency signals to the consumable chips. These elastomer pins have low losses and a low parasitic series inductance in a compressed state up to 50 GHz according to the manufacturer [20].

The mechanical part of the system consists of a spring-levered system with a handle, used for fixing the consumable fluidic chip to the Invisipins on the PCB, and also for providing enough pressure to compress the Invisipins to the conducting state (meaning a minimal contact resistance) (Figure 1b). During the microwave-microfluidic chip loading procedure, the springs are maximally compressed by manually pushing the handle, as shown in Figure 1d,e. This is followed by inserting the chip through a slit into the enclosure, just above the conducting elastomer Invisipins, depicted in (Figure 1f,g). Once the chip is correctly positioned, the handle is released and both electrical and mechanical contacts are achieved. Moreover, due to the sufficient pressure, a stable mechanical positioning is obtained and the chip stays fixed on this location throughout measurement. Finally, the correct loading of a chip on the setup is only a matter of seconds as the fluidic chip and the slip are closely sized (Figure 1c).

Aside from the main purpose of the PMMA enclosure to house electric–mechanic system, its secondary function is to protect the microwave transmission line from environmental influences such as moisture in the incubator, proximity of metal wires that influence the signal propagation over the line, and so on. Therefore, the PMMA enclosure has only two openings, one for the SMA connector and the other slit for loading the chip, as shown in (Figure 1b).

### 2.2. Electrical Characteristics

**Figure 2:**
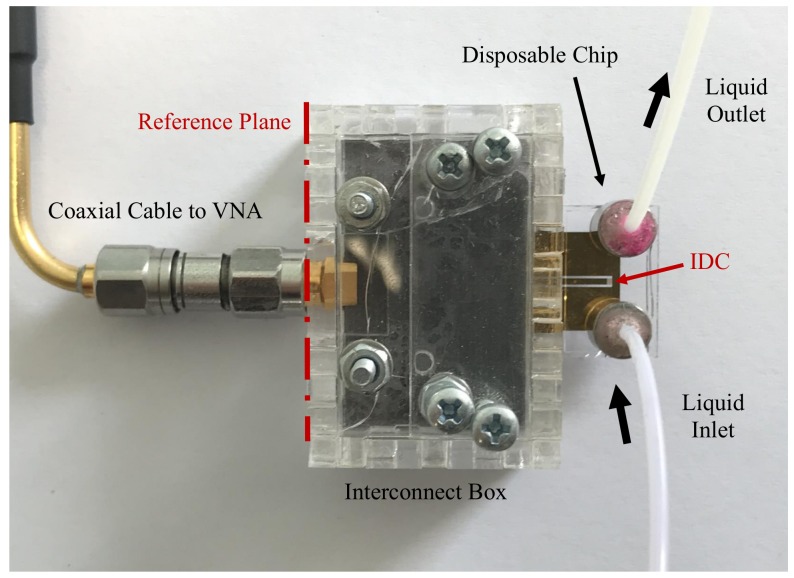
Photograph of the measurement setup with the quartz consumable chip mounted in the ICB.

**Figure 3:**
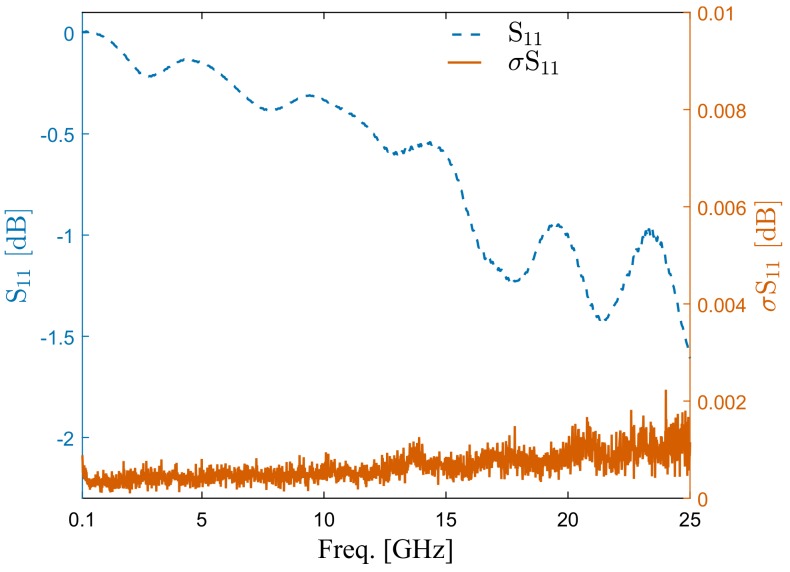
Measured reflection coefficient (S${}_{11}$) of the unloaded ICB and the standard deviation between several measurements in dB.

The ICB adds an extra transmission line to the measurement setup, which adds an extra insertion loss and phase delay to the complete setup. The reflection coefficient of the ICB without loading a chip was measured to estimate the insertion loss of the ICB. The measured reflection coefficient of an ideal ICB should be 1 or 0 dB, since it acts as an open-ended transmission line that reflects back all input power. The measurement setup, presented in Figure 2, shows the ICB directly connected to VNA using a coaxial cable. A one-port short open load thru (SOLT) calibration is performed to move the reference plane to the end of the coaxial cable that will be connected to the SMA connector of the ICB. The measurements were repeated 6 times, and the average magnitude of the reflection coefficient and the standard deviation between different measurements were calculated from 100 MHz to 25 GHz, as shown in Figure 3. The measured reflection coefficient of the ICB is larger than –0.5 dB up to around 12 GHz, and it increases with the frequency since the CPW transmission line on PCB has larger loss at higher frequencies. The reflection coefficient of the ICB is larger than –1.6 dB up to 25 GHz. This indicates that the ICB does not add much loss to the system and can perform well for microwave-microfluidic applications up to 25 GHz.

## 3. Application for Microwave-Microfluidic Sensors

The proposed ICB is used as the interface between the VNA and IDC-based microwave-microfluidic sensors—with the duty to bring the microwave signal from the VNA through a coaxial cable to the CPW transmission line on the quartz chip. This proves its potential to be used as a reliable, instantly reconnectable interface to bring the microwave signals to the lab-on-a-chip system. Traditionally, those measurements are performed with either a probe-based on-wafer approach or a connector-based approach, which have certain drawbacks as discussed in the previous section of this paper. The IDC-based microwave-microfluidic sensor consists of a patterned gold layer to achieve an IDC embedded in a microfluidic channel fed by a CPW transmission line.

### 3.1. Contact Stability and Repeatability

The stability of the measurement is important for microwave-microfluidic applications. It directly decides the minimum distinguishable difference of S-parameters in sensing applications and the uncertainty of the extract electrical permittivity in broadband spectroscopy applications. The stability of measurements depends on several facts, such as the noise of the measurement instrument, the stability of the contacting, and the stability of the measurement environment—such as the temperature of the environment and the mechanical stability of the measurement setup. The stability and repeatability of the contact between the Invisipins on PCB in ICB and the quartz chip is investigated by microwave measurements. An IDC sensor chip was inserted into the ICB of the measurement setup shown in Figure 2. The cable and ICB were fixed to the measurement table to minimize their possible movement and consequently enhance the mechanical stability of the measurement setup. The temperature in the laboratory was controlled by air conditioner. Furthermore, the microfluidic channel was kept empty over measurements, since the dielectric permittivity of the loaded liquid is more sensitive to the temperature change than air. Thus, the possible influence of the temperature change on the measurement results is minimized. The standard deviation of S${}_{11}$ between different measurements of the ICB shown in Figure 3 gives an educated indication of the measurement uncertainty caused by the noise of the VNA, since the SMA connector assure a very stable connection between the VNA and ICB.

**Figure 4:**
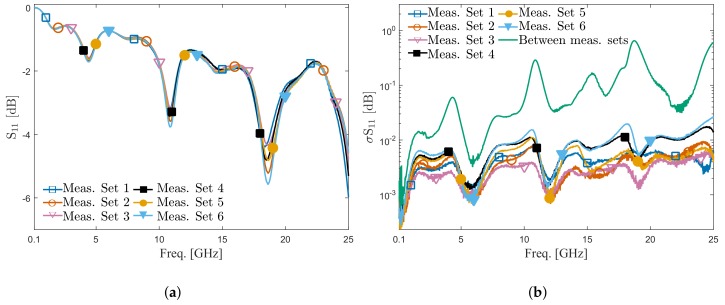
Measurement results of reflection coefficient (S_11_) with consumable interdigital capacitor (IDC) sensors with the microfluidic channel loaded with air for different measurement sets. (**a**) Mean value for each measurement sets. (**b**) Standard deviation within the same measurement sets and between the mean value of different measurement sets.

The stability and repeatability of the contact between the Invisipins and gold on the quartz chip was evaluated with the following steps. First, a quartz chip was inserted and a contact between the Invisipins and gold on the quartz chip was obtained. A set of ten measurements of reflection coefficient was performed while maintaining the chip position. The mean value and standard deviation of these ten measurement results—defined as measurement set one—were calculated. The standard deviation in this case reflects the stability of the contact between the Invisipins and gold on the quartz chip. In the second step, the chip was taken out of the ICB and inserted back into the ICB. Once the contact between the Invisipins and gold was obtained, a new set of ten measurements was performed. Once again, the mean value and standard deviation were calculated for measurement set two. These procedures were repeated six times to get six sets of measurement results. Finally, the standard deviation between the mean value of the measured reflection coefficient of the six measurement sets was calculated and used to investigate the contact repeatability over different measurement campaigns. The mean magnitudes of measured S${}_{11}$ of different measurement sets are shown in Figure 4a. The ICB loaded with a quartz chip has higher insertion loss than the unloaded ICB over the whole frequency band since the quartz chip and the Invisipins introduce additional losses in the signal path. A difference of measured S${}_{11}$ between different contacts can be observed in a high-frequency range. It is caused by a slight change of the manually obtained physical position of the inserted quartz chips into the ICB over different measurement repetitions. Figure 4b shows the standard deviation corresponding to individual measurement sets, and finally, the standard deviation of mean values corresponding to individual measurement sets. As can be seen from data in Figure 4b, the influence of manual positioning of the chip between measurement repetitions starts to have impact at higher frequencies. This can be expected as the influence of the chip misalignment at higher frequencies is more pronounced due to the increase of the interconnect electrical size with frequency. In contrast, for the measurement results within the same measurement set, the chip’s physical position in the ICB is fixed and the standard deviation is low. In summary, the standard deviation varies between different contacts and up to 10 GHz is smaller than 0.1 dB, which indicates that the repeatability of the contact is not as good as on-wafer measurements. Nevertheless, given the price of this setup and aimed applications, the proposed ICB can be confidently used as a stable interface between the VNA and the microwave-microfluidic chips. Further improvement of the ICB can be made in the view of mechanical stoppers that will fix the physical position of the quartz chip, finally improving the repeatability of the contact.

### 3.2. Quartz Chip Design

IDC sensors for microwave-microfluidic applications were designed and fabricated on a 4-inch quartz wafer. The fabrication process of the IDC sensor chips consists of three main steps [10]. First, a lift-off process is performed to pattern a 400-nm-thick gold layer following a titanium-tungsten adhesion layer to build IDC structures on quartz wafer. Then, a 20-μm-thick microfluidic channel is built in a PDMS layer to guide liquid flow over the IDC sensor. Finally, the wafer is diced into single chips with compatible dimensions to be inserted into the proposed ICB.

The capacitance of on-chip IDCs depends on the physical dimensions of its electrodes and the dielectric permittivity of both the substrate and material covering the electrodes. By guiding the liquid under test on top of the electrodes through microfluidic channel in a PDMS layer, the capacitance of the IDC is changed, and this change will be detected by a microwave measurement instrument. A method has been proposed to extract electrical permittivity of the liquid under test with an on-wafer measurement setup to achieve a broadband spectroscopy [21]. This method requires on-wafer calibration and precise characterization of the CPW transmission line to move the reference plane to the IDC. The dielectric permittivity can be extracted through a precise model of the used IDC. However, this method can not be applied to the ICB-based measurement setup since the repeatability of the contacting is not as high as an on-wafer measurement setup. Therefore, the ICB box should not be calibrated by loading different standard chips. Nonetheless, a permittivity extraction method using an open coaxial probe was proposed by Wei [22,23], which can be applied in our case. By measuring the reflection coefficients of the sensor loaded with three different liquids with known electrical permittivity, the electrical permittivity of the unknown liquid can be calculated with the following equation:

(1) $$\frac{\left(\epsilon_{M}-\epsilon_{A}\right)\times \left(\epsilon_{B}-\epsilon_{C}\right)}{\left(\epsilon_{M}-\epsilon_{B}\right)\times \left(\epsilon_{C}-\epsilon_{A}\right)}=\frac{\left(\Gamma_{M}-\Gamma_{A}\right)\times \left(\Gamma_{B}-\Gamma_{C}\right)}{\left(\Gamma_{M}-\Gamma_{B}\right)\times \left(\Gamma_{C}-\Gamma_{A}\right)}.$$

as shown in Equation (1), $\Gamma_{A}$, $\Gamma_{B}$, $\Gamma_{C}$, and $\Gamma_{M}$ represent the measured reflection coefficients of the device at the reference plane shown in Figure 2. These reflection coefficients correspond to a sensors loaded with three different reference liquids with the known permittivity of $\epsilon_{A}$, $\epsilon_{B}$, and $\epsilon_{C}$, and the permittivity $\epsilon_{M}$ of the liquid under test, respectively. The key point of this method is that the capacitance changed by the loaded liquid should be linear to the dielectric permittivity of the loaded liquid. The rest of the setup is considered to be constant for the whole measurement procedure. In other words, since this method eliminates the necessity to characterize the measurement setup prior to flushing liquids, it was applied on our ICB measurement setup for extraction of dielectric permittivity of loading liquids.

**Figure 5:**
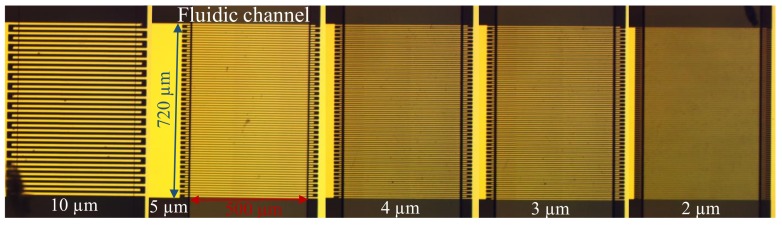
Microscope photograph of the fabricated IDC sensors with different dimensions of electrode width and gap width between electrodes.

Capacitance of on-chip IDC built with interdigital electrodes [24,25] or conductive metal–organic framework (MOF) electrodes [26] using multilayered [24,26] or a metal-oxide-semiconductor (MOS) structure [25] have been investigated and modeled. According to the IDC model proposed by Igrela [24], the capacitance of the planar IDC is linearly proportional to the dielectric permittivity of the material loaded on the electrodes of the IDC. This factor depends on the length of the electrodes, the metallization ratio (the ratio between the width of an individual electrode and the gap between the electrodes), the number of electrodes, and the thickness of the loaded materials. Although the exact width of an individual electrode and the gap between the electrodes does not appear in the model, for an IDC with a certain total width, the narrower the individual electrodes are, the more electrodes it contains, which help to achieve a larger capacitance change for given dielectric permittivity change. In this paper, five IDC sensors with the same physical dimensions but different electrode widths were designed. The metallization ratio is fixed to 0.5, which means the width of the electrodes is identical to the gap between the electrodes. The width of the electrodes are set to 2 μm, 3 μm, 4 μm, 5 μm, and 10 μm, and the total width of the IDC is 720 μm. As shown in Figure 5, the PDMS wall covered the edge of the electrodes, which introduced an arbitrary fringing field. The width and height of the microfluidic channel is 500 μm and 20 μm, respectively. These IDC sensors have an identical sensing area of 0.36 mm${}^{2}$ and sensing volume of 7.2 nL. The capacitance change caused by unit relative electrical permittivity is 78.5 fF, 158.2 fF, 198.1 fF, 264.5 fF, and 397.3 fF for the IDC with an electrode width of 10 μm, 5 μm, 4 μm, 3 μm, and 2 μm, respectively. The feeding CPW transmission line for the IDC sensors has a central conductor width of 720 μm and ground width of 5000 μm. The gap between the central conductor and the ground is 260 μm. The transmission line on a quartz wafer was sized to match the transmission line on the PCB located within the ICB.

### 3.3. Measurement Results and Permittivity Extraction

With the proposed ICB, the fabricated chips can be easily and rapidly used in measurements. As shown in Figure 2, the microfluidic channel on the sensor chip is connected to the fluidic pump with a magnetic interface [27]. After the magnets were used to fix the tube to the sensor chip, the chip was inserted and clipped to the ICB. During the measurement, the setup was untouched, and liquid mixtures of IPA and DI water were made with the volume fraction of IPA 0%, 20%, 40%, 60%, 80%, and 100%. The IPA/DI water mixtures are chosen because the electrical permittivity of the mixtures are well modeled [28] and those values can be used to achieve a permittivity extraction.

**Figure 6:**
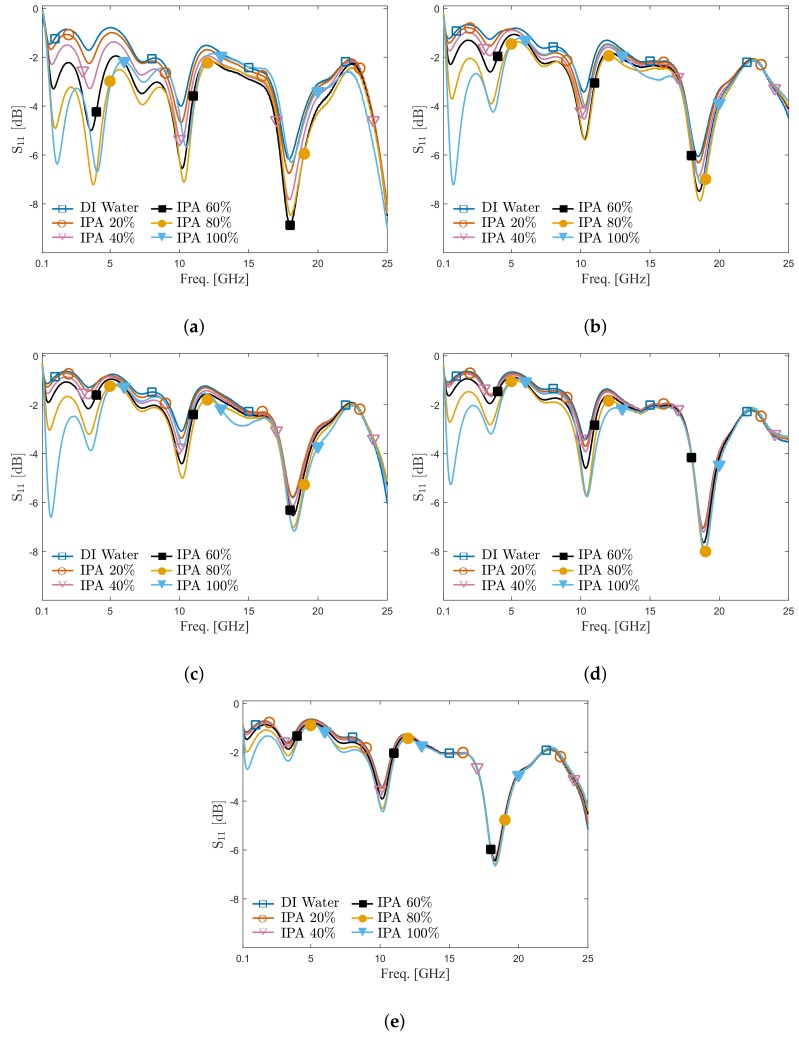
Measured reflection coefficient S_11_ for the mixture of deionized (DI) water and Isopropanol (IPA) with different concentrations and with IDCs having different electrode widths and separation between electrodes. Electrode width and separation between electrode both (**a**) 10 µm, (**b**) 5 µm, (**c**) 4 µm, (**d**) 3 µm, (**e**) 2 µm.

The magnitude of the reflection coefficient is measured from 100 MHz to 25 GHz for different IDC sensors, as shown in Figure 6. The selected frequency band is limited by the minimum operating frequency of the used VNA and the maximum operating frequency of the used SMA connector in the ICB. Relaxation frequencies of solutions having biomolecules of interest, such as glucose [29] and various of amino acids [30], are located in this frequency band. In all subfigures in Figure 6, the minimum and maximum of measured reflection coefficients occur around similar frequencies because the reference plane during measurements is at the connection of the ICB SMA connector and the VNA, as shown in Figure 2. This means the contribution of the ICB, the contact between the ICB and the quartz chip, and the CPW transmission line on the quartz chip are not eliminated from measurement data. Furthermore, at all these discontinuities, reflections take place just like in any microwave measurement setup and consequently influence the measured reflection coefficient at the reference plane. Nevertheless, for all chips accommodating different IDC designs which are inserted into the ICB, the contribution of the ICB, the connection between the ICB and the quartz chip, and the CPW transmission line on a quartz wafer are consistent, as can be seen from the measurement data in Figure 6. Despite these effects, the liquid mixtures having different volume fractions can still be distinguished using five proposed IDC sensors in the frequency band, as shown in Figure 6.

**Figure 7:**
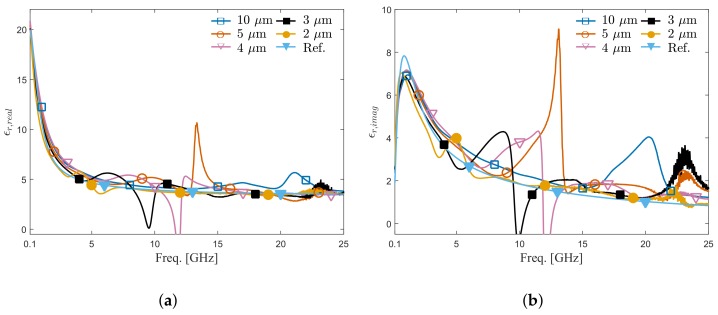
Extracted permittivity using IDCs with different electrode widths compared with reference permittivity for DI water/IPA Mixture with IPA volume fraction 80%. (**a**) Real part. (**b**) Imaginary part.

**Figure 8:**
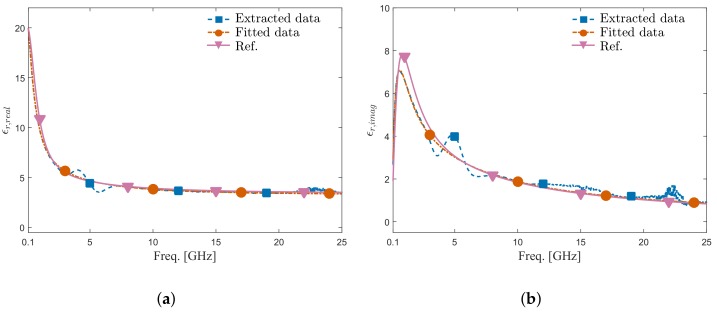
Extracted and fitted permittivity using IDC with an electrode width of 2 µm compared with reference permittivity for DI water/IPA Mixture with IPA volume fraction 80%. (**a**) Real part. (**b**) Imaginary part.

By using Equation (1), the dielectric permittivity of mixture with 80% IPA volume fraction was extracted using the measured reflection coefficients and modeled dielectric permittivity (from [28]) of the mixtures with IPA volume fractions of 40%, 60%, and 100%. The real and imaginary part of the extracted dielectric permittivity for all five different IDCs and the data calculated based on the model from Reference [28] are shown in Figure 7a,b, respectively. The results show that at frequencies below 1 GHz, all IDC sensors provide results close to the values given by the model. However, as the frequency increases, the extracted values start to deviate from the model value due to the mathematical instability of Equation (1) [31] for small signal changes coming from sensors. In other words, as the electrode width gets smaller, the electrodes get closer and the dielectric material has a stronger influence on the capacitance value, which finally results in larger measured signal changes. In that manner, it can be said that the IDC having the electrode width of 2 μm gives the extracted permittivity closest to the reference data, which is confirmed by measurement results shown in Figure 7. The extracted permittivity using IDC with an electrode width of 2 μm was fitted to the model from Reference [28]. As shown in Figure 8, the fitted permittivity agrees well with the reference data calculated based on the model from Reference [28]. In summary, even though the proposed ICB introduced a transmission line that is challenging to de-embed using traditional calibration methods, the proposed interconnect platform and IDC sensors achieves permittivity extraction with satisfying accuracy in the frequency range from 100 MHz to 25 GHz.

## 4. Conclusions

A novel microwave platform for biosensing conceived around the interconnect box is proposed to bring microwave signals to microwave-microfluidic chips with a verified stable contact. In addition to the microwave platform, a set of consumable microwave-microfluidic chips was designed based on the interdigital electrode array topology to test the microwave platform. The IDCs having different capacitances over the identical sensing area were loaded onto the proposed microwave platform and measured with VNA. The IDC sensor designed with the largest capacitance offers broadband spectroscopy in the frequency range from 100 MHz to 25 GHz. Further research will focus on increasing the permittivity extraction accuracy by improving alignment procedures for consumable chips and incorporating temperature stability. By avoiding the usage of the bulky, immobile, and expensive probe station—and extra costs related to connectorization of disposal chips—microwave-microfluidics sensing and heating devices are getting an opportunity to enter life science laboratories and make an impact in interdisciplinary biosensing domains.

## Abbreviations

The following abbreviations are used in this manuscript:

CPW	co-planar waveguide
DI	deionized
HUVEC	human umbilical vein endothelial cells
ICB	interconnect box
IDC	interdigital capacitor
IPA	isopropanol
MOF	metal–organic frameworks
MOS	metal-oxide-semiconductor
PCB	printed circuit board
PDMS	polydimethysiloxane
PMMA	polymethylmethacrylate
SIW	substrate integrated waveguide
SMA	subminiature type A
SOLT	short open load thru
VNA	vector network analyzer
